# Association between Crash Attributes and Drivers’ Crash Involvement: A Study Based on Police-Reported Crash Data

**DOI:** 10.3390/ijerph17239020

**Published:** 2020-12-03

**Authors:** Guofa Li, Weijian Lai, Xingda Qu

**Affiliations:** Institute of Human Factors and Ergonomics, College of Mechatronics and Control Engineering, Shenzhen University, Shenzhen 518060, China; guofali@szu.edu.cn (G.L.); laiweijian2018@email.szu.edu.cn (W.L.)

**Keywords:** driving safety, driver age, crash involvement, logistic regression

## Abstract

Understanding the association between crash attributes and drivers’ crash involvement in different types of crashes can help figure out the causation of crashes. The aim of this study was to examine the involvement in different types of crashes for drivers from different age groups, by using the police-reported crash data from 2014 to 2016 in Shenzhen, China. A synthetic minority oversampling technique (SMOTE) together with edited nearest neighbors (ENN) were used to solve the data imbalance problem caused by the lack of crash records of older drivers. Logistic regression was utilized to estimate the probability of a certain type of crashes, and odds ratios that were calculated based on the logistic regression results were used to quantify the association between crash attributes and drivers’ crash involvement in different types of crashes. Results showed that drivers’ involvement patterns in different crash types were affected by different factors, and the involvement patterns differed among the examined age groups. Knowledge generated from the present study could help improve the development of countermeasures for driving safety enhancement.

## 1. Introduction

Road traffic crashes are a major challenge to public health [1,2]. According to a recent report from the World Health Organization, the number of deaths in road crashes remains unacceptably high, with an estimation of 1.35 million each year [3]. Various countermeasures (e.g., roadside facilities) have been proposed to reduce or mitigate traffic crashes [4,5]. In order to design effective countermeasures for driving safety improvement, a better understanding of the factors influencing drivers’ crash involvement becomes necessary [6].

A wealth of crash-related studies have assessed driver characteristics (e.g., age) that are associated with elevated crash involvement. With a steadily aging population worldwide, age has long been recognized as a critical influencing factor in crashes [7]. Previous studies showed that younger male drivers and older drivers were more susceptible to crash involvement [8], and crash statistics supported this conclusion [9]. As compared to middle-aged experienced drivers, younger drivers have higher violation rates, tend to underestimate the risks of various violations, have a lower level of motivation to follow traffic rules, and are overly involved in running red lights [7,10,11]. These injudicious and risk-taking behaviors are closely associated with increased crash risk [12]. Unlike younger drivers, the crash risks among older drivers can be attributed to their functional decline in vision, attention, and decision making [13]. Meanwhile, older drivers experience greater mental workloads than younger drivers due to their age-related decline in cognitive capabilities [14].

Besides driver characteristics, unsafe driver behaviors such as speeding, distraction, and driving under the influence (DUI, i.e., drunk and drugged driving) will also make drivers more likely to be involved in crashes. Previous studies reported that speeding is one of the primary causes of road crashes, leading to 26% of all crash fatalities in the U.S. in 2017 [9]. Distracted driving caused by cellphone use also contributes greatly to crash risk and has become a prominent issue because of the overwhelming increase in the use of smartphones and in-vehicle entertainment devices [15]. About 71% of young drivers who were killed in road crashes were reported to have experience of message texting while driving [9]. Therefore, many countries (e.g., U.S., Canada, China) have banned message texting and even hand-hold use of a cellphone while driving, but still many other potential uses of cellphones (e.g., hands-free calling) have not yet been legislated. The use of alcohol or drugs is also severely harmful to driving safety. About 11,000 deaths are caused by alcohol-impaired driving every year in the U.S., accounting for 29% of all traffic-related fatalities in 2017 [9].

Moreover, environmental factors (e.g., weather and time of day) could also affect drivers’ crash involvement. Based on a three-year crash dataset in the south-central area of the U.S., the authors of [16] found that drivers were more likely to be involved in crashes on rainy days. A study based on the Fatality Analysis Reporting System (FARS) data reported that fatal crashes in rain were three times as likely to involve ≥10 vehicles as fatal crashes on clear days [17]. Nighttime driving is also dangerous with adjusted fatality rates being up to three times higher than daytime driving [18]. This situation is even worse for fatal crashes involving pedestrians, where pedestrian fatalities at night occur at higher rates compared to pedestrian fatalities during the day [19], and its rate is up to seven times higher than that in daytime [20]. Dozza [21] analyzed the data from 11 roadside stations in Gothenburg and found that crash risk was greatest at night on weekends.

There are different types of crashes and the causations may differ across the crash types. Understanding the association between crash attributes and drivers’ crash involvement in different types of crashes can help figure out the causation of crashes, and further aid in developing effective countermeasures for crash avoidance. However, this knowledge is quite lacking in the literature. To fill this research gap, the aim of this study was to examine the involvement in different types of crashes for drivers from different age groups, by using the police-reported crash data from 2014 to 2016 in Shenzhen, China. Crash attributes mainly defined by driver characteristics and environmental factors were recorded in the selected dataset. A synthetic minority oversampling technique (SMOTE) together with edited nearest neighbors (ENN) were used to solve the data imbalance problem caused by the lack of crash records of older drivers. Logistic regression was utilized to estimate the probability of a certain type of crash, and odds ratios that were calculated based on the logistic regression results were used to quantify the association between crash attributes and drivers’ crash involvement in different types of crashes.

The main contribution of this study is that it examined crash involvement of drivers from different age groups in different crash types. This extends the previous work from a single factor analysis or a mixed crash-type analysis to an analysis on the influencing factors. This study also provides an insight into Chinese traffic safety facts. To the best of our knowledge, the present study is one of the first attempts to examine drivers’ crash involvement in China by considering driver characteristics, environment factors, and crash types. The work presented in this study would help design countermeasures for traffic safety enhancement.

## 2. Materials and Methods

### 2.1. Traffic Crash Data

This study was based on a 3-year (2014−2016) dataset of police-reported traffic crashes in Shenzhen, China. The data were obtained from the Information Sharing Platform for Road Traffic Safety Research in China. In total, 237,255 crashes were reported during the 3 years. Attributes of crashes including day of the week, time of day, weather, road type, vehicle type, driver gender and age were recorded. Three age groups, corresponding to younger drivers (18−30 years), middle-aged drivers (40−50 years), and older drivers (>60 years), were extracted from the dataset for analysis. Note that drivers aged from 31 to 39 and 51 to 59 were excluded in order to better reveal the age effect on drivers’ crash involvement. As for the other factors, time of day was equally segmented into four segments including 0:00~5:59 (denoted as 0~5), 6:00~11:59 (6~11), 12:00~17:59 (12~17), and 18:00~23:59 (18~23), with 6 h in each. The road types were also divided into three groups according to the speed limit, including low-speed limit roads (≤0 km/h), medium-speed limit roads (30 km/h~60 km/h), and high-speed limit roads (≥60 km/h). Table 1 presents the recorded crash attributes for analysis. The number of crashes with full records of all the attributes shown in Table 1 is 72,238.

In total, 23 types of crashes were reported, similar to the crash types defined in [9]. In general, among the reported crash types, the top five associated with the highest numbers of crashes were crashes with motor vehicles in transport (CMVT, e.g., rear-end, head-on, and intersection crashes with moving vehicles), crashes with stopped vehicles (CSV), other crashes between vehicles (OCV, e.g., crashes between motor vehicles and nonmotor vehicles), sideswipe crashes with pedestrians (SCP), and crashes with fixed objects (CFO, e.g., crashes with roadside facilities), accounting for 98.50% of all the crashes. Therefore, only the top five crash types were analyzed in this study.

### 2.2. Synthetic Minority Oversampling Technique (SMOTE) and Edited Nearest Neighbors (ENN)

**Figure 1:**
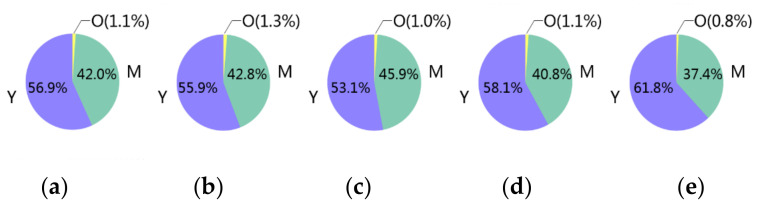
Age distribution in the five examined crash types. Y: younger, M: middle-aged, O: older. (**a**) CMVT: crashes with motor vehicles in transport, (**b**) CSV: crashes with stopped vehicles, (**c**) OCV: other crashes between vehicles (e.g., crashes between motor vehicles and nonmotor vehicles), (**d**) SCP: sideswipe crashes with pedestrians, (**e**) CFO: crashes with fixed objects.

Figure 1 shows the age distribution in the five examined crash types. The percentages of older drivers in crashes with motor vehicles (CMVT), crashes with stopped vehicles (CSV), other crashes between vehicles (OCV), sideswipe crashes with pedestrians (SCP), and crashes with fixed objects (CFO) were 1.1%, 1.3%, 1.0%, 1.1%, 0.8%, respectively. The number of older drivers was far less than the number of younger or middle-aged drivers in each crash type. See Table 2 for the exact numbers of each age group in each crash type. The extremely imbalanced sample numbers across age groups would cause invalid developed models [22,23,24]. In addition, analyzing the characteristics of older drivers is urgently needed for traffic safety enhancement given the aging population in China [25]. Batista et al. compared 15 data manipulation techniques and found that synthetic minority oversampling technique (SMOTE) together with edited nearest neighbors (ENN) outperformed the other methods in terms of dealing with imbalanced data [26]. Hence, SMOTE + ENN were used in this study to solve the imbalance problem.

SMOTE is an upsampling method which produces new samples for minority classes by interpolating between the samples that lie together [26,27]. It works by selecting samples that are close to each other in the feature space, drawing a line between the samples in the feature space, and then generating a new sample at a point along that line. Specifically, a target sample from the minority class is randomly chosen at first. Then, the nearest *k* neighbors of that sample can be determined (typically *k* = 5). A random neighbor is then selected from the *k* neighbors and a synthetic sample is randomly created along the line between the target point and the selected neighbor in the feature space. This procedure can be used to create as many synthetic samples for the minority class as possible.

ENN is a downsampling method to remove the samples whose class label differs from the majority of its *k* nearest neighbors [26,28]. Specifically, the majority is usually defined as more than half of the *k* nearest neighbors [26,28]. As suggested in [26], *k* = 5 was applied in the present study. By applying SMOTE to upsample the older and middle-aged driver groups and then using ENN to downsample all the age groups, the dataset could be balanced across different age groups. Algorithm 1 shows the pseudocode of SMOTE + ENN. The source code of SMOTE + ENN can be found at: https://github.com/scikit-learn-contrib/imbalanced-learn.

**Algorithm 1:** Pseudocode of the SMOTE+ENN algorithm
1:**Input:** Imbalanced dataset *S*; Numbers of nearest neighbors *k*2:**Output:** Processed dataset *S*3:  **for** each point *p* in *S* **do**4:      compute its *k* nearest neighbors in *S*.5:      randomly choose *r* ≤ *k* of the neighbors6:  choose a random point along the lines joining *p* and each of the *r* selected neighbors.7:    add these synthetic points to the dataset with class *S*.8:  **end for**9:  **for** each point p in S **do**10:    compute its *k* nearest neighbors in *S*.11:    **if** more than half of the neighbors are different from label of *p* **then**12:      remove *p* from *S*.13: **end for**14: **return** *S*


### 2.3. Relationship Between Crash Attributes and Drivers’ Crash Involvement

Based on the balanced dataset, we utilized a logistic regression approach to estimate the relationship between the examined crash attributes and crash involvement among different age groups. Wald test was used to determine the statistical significance of the explanatory variables. The selected crash types were analyzed separately. The response variable was set at 1 when the target crash type occurred, and was set at 0 for the cases of the other crash types. The binary logistic regression formula can be expressed as:

(1) $$P\left(y=1|x\right)=\frac{1}{1+e^{-\left(\beta_{0}+\beta_{1}x_{1}+\ldots +\beta_{n}x_{n}\right)}}$$

where *P*(*y* = 1|***x***) is the probability of the target crash type; **x** is the vector of the explanatory variables (*x*_1_, *x*_2_, …, *x_n_*) that are defined by the crash attributes as presented in Table 1; *β*_0_ is a constant and *β*_1_, *β*_2_, ⋯, *β_n_* are the coefficients of explanatory variables. The expected probability of *y* = 0 can be calculated as:

(2) $$P\left(y=0|x\right)=1-\;P\left(y=1|x\right)=\frac{1}{1+e^{\beta_{0}+\beta_{1}x_{1}+\ldots +\beta_{n}x_{n}}}$$

Odds ratio (OR) is a statistic that quantifies the association strength between exposures and outcomes, and it has been frequently reported in the studies using traffic crash data to understand crash causations [29,30]. In this study, OR was calculated to reflect the association between crash attribute and drivers’ involvement in the target crash type. The reference of each attribute was defined in Table 1. The equations to calculate the odds of drivers’ involvement in the target crash type is given in Equation (3), as follows:

(3) $$\mathrm{odds}=\frac{P\left(y=1|x\right)}{P\left(y=0|x\right)}=e^{\beta_{0}+\beta_{1}x_{1}+\ldots +\beta_{n}x_{n}}$$

The selected crash attributes are all categorical variables, so we generated dummy variables to calculate OR using the method suggested by [31]. The dummy variable d_i_ of the *i*-th attribute is defined in Equation (4). In this study, we have 7 attributes in total (age, weather, gender, time of day, day of the week, vehicle type, and road type), hence *i* = 1, 2, ⋯, 7. *m* is the number of discrete status values of the *i*-th attribute and the dimension of the dummy variable $d_{i}$ equals to *m* − 1. For example, the number of discrete status values of vehicle type (*i* = 6) is 4 and the dummy variable *d*_6_ is *d*_6_ = ($d_{6}^{2}$, $d_{6}^{3}$, $d_{6}^{4}$).

(4) $$d_{i}=\left(d_{i}^{2},\;d_{i}^{3},..,d_{i}^{m}\right)$$

where the dummy variable subset $d_{i}^{j}$ for the *j-*th status value of the *i*-th attribute is defined in Equation (5). The reference attribute status (e.g., car in vehicle type) corresponds to *j* = 1 and all the m − 1 corresponding values in $d_{i}^{1}$ are all 0. The other attribute status is set as 1 on its corresponding position and 0 on other positions. For example, the dummy variable subset of truck (*j* = 3) is $d_{6}^{3}$ = [0, 1, 0].

(5) $$\begin{array}{c} d_{i}^{j}=\left\{\begin{array}{c} \left[x_{i}^{2}=0,\;x_{i}^{3}=0,...,\;x_{i}^{j}=1,..,x_{i}^{m}=0\right]\;\;\;\;,j\neq 1\;\\ \;\;\;\;\;\;\;\;\;\;\;\;\;\;\;\;\;\;\;\;\;\;\;\left[0,...,0\right]\;\;\;\;,\;j=1 \end{array}\right. \end{array}$$

For the *i*-th attribute, the calculation of OR for the *j-*th discrete attribute status based on the generated dummy variables is given in Equation (6). The OR($x_{i}^{j}$) uses $d_{i}^{1}\;$ as the reference attribute status and calculates the influence of $d_{i}^{j}\;$ as:

(6) $$\mathrm{OR}\left(x_{i}^{j}\right)=\frac{\mathrm{odds}\left(d_{i}^{j}\right)}{\mathrm{odds}\left(d_{i}^{1}\right)}=\frac{e^{\beta_{0}+\beta_{1}x_{1}\ldots +\beta_{i}d_{i}^{j}+\ldots \beta_{n}x_{n}}}{e^{\beta_{0}+\beta_{1}x_{1}\ldots +\beta_{i}d_{i}^{1}+\ldots \beta_{n}x_{n}}}=e^{\beta_{i}^{j}}$$

(7) $$\beta_{i}=\left[\beta_{i}^{2},\;\beta_{i}^{3},..,\beta_{i}^{m}\right]$$

where $\beta_{i}^{j}$ is the coefficients of $x_{i}^{j}$ in $d_{j}^{i}$. OR = 1 means that when compared to the reference attribute status, the attribute status $x_{i}$ does not affect the probability of drivers’ involvement in the target crash type, OR > 1 means the attribute status $x_{i}$ will increase the probability, and OR < 1 means the specific attribute *x_i_* status will reduce the probability.

We used Python 3.6 (Python Software Foundation, Delaware, United States) for the SMOTE + ENN, IBM SPSS Statistics 22.0 (IBM, Armonk, NY, USA) for the logistic regression analysis, and MATLAB R2018a (MathWorks, Natick, MA, USA) for data cleaning, feature extraction and visualization in this study.

## 3. Results

### 3.1. Association Between Crash Attributes and Younger Drivers’ Crash Involvement

Table 3 shows the OR results for younger drivers. It was found that most crash types occurred more on sunny days than on rainy days except CFO (OR = 2.16, *p* < 0.001). The ORs for OCV and SCP crashes were lower than 0.5 for female drivers, while the OR for CMVT crashes was 1.41 (*p* < 0.001). As for the influence of time of day, the OR for CMVT crashes was the highest during the time period of 12−17 pm (OR = 4.30, *p* < 0.001), while the OR for CSV crashes during the time period of 0−5 am was much higher (OR = 10.13, *p* < 0.001) than all the other time periods. SCP crashes occurred more frequently during the time period of 18−23 pm (OR = 1.72, *p* = 0.001) than the other time periods, and the lowest OR was observed during 12−17 pm (OR = 0.23, *p* < 0.001). The ORs for CFO crashes during 12−17 pm and 18−23 pm. were significantly lower than the reference time period (6−11 am). The ORs for CMVT and SCP crashes on Friday were all significantly higher than the reference day of the week (Monday), while the ORs for the other three crashes were all significantly lower than the reference Monday. Considering vehicle type influence, the ORs for CMVT crashes were significantly higher with buses and trucks, while the ORs for OCV crashes with buses and trucks were lower (*p* < 0.05) than those with passenger cars. As for the road type influence, the ORs for CFO crashes on medium- and high-speed limit roads were significantly higher than that on low-speed limit roads, while the ORs for CMVT and CSV crashes on medium- or high-speed limit roads were lower than those on low-speed limit roads.

### 3.2. Association Between Crash Attributes and Middle-Aged Drivers’ Crash Involvement

The results presented in Table 4 show that middle-aged drivers were more likely to be involved in OCV crashes (OR = 1.47, *p* = 0.005), SCP crashes (OR = 1.30, *p* = 0.011), and CFO crashes (OR = 2.64, *p* < 0.001) on rainy days than on sunny days, but less likely to be involved in CMVT crashes (OR = 0.41, *p* < 0.001). Female middle-aged drivers were more likely to be involved in CMVT crashes (OR = 1.80, *p* < 0.001), but less likely to be involved in the other types of crashes. The time of day results show that the ORs for CMVT crashes during 12−17 pm and 18−23 pm were higher than the reference time period (6−11 am). The ORs for OCV and SCP crashes during 18−23 pm and 0−5 am were all lower than the reference time period, while the ORs for SCV crashes during these two time periods were higher than the reference time period. However, with younger drivers, almost all the ORs from Tuesday to Sunday were significantly lower than those on Monday for middle-aged drivers involved in OCV, SCP, and CFO crashes, but the trend was opposite for CMVT crashes. Similarly with younger drivers, the ORs for CSV and OCV crashes with buses or trucks were all significantly lower than those with passenger cars, but the ORs for CMVT crashes were higher with buses (OR = 1.90, *p* < 0.001) and trucks (OR = 1.49, *p* < 0.001). The ORs for CSV and SCP crashes on medium and high-speed limit roads were all significantly lower than on low-speed limit roads, while the OR for CMVT crashes on high-speed limit roads was higher (OR = 3.70, *p* < 0.001) than on the reference low-speed limit roads. 

### 3.3. Association between Crash Attributes and Older Drivers’ Crash Involvement

The results in Table 5 show that on rainy days older drivers were much more likely to be involved in CMVT crashes (OR = 67.62, *p* < 0.001), but less likely to be involved in CSV, SCP, and CFO crashes. The OR of older female drivers involved in CMVT crashes was 60.47 with statistical significance (*p* < 0.001), while the number was 0.03 for CFO crashes. An interesting result on the influence of time of day on older drivers was that almost all the ORs during 18−23 pm were different to the trends of ORs during 12−17 pm in the examined CMVT, CSV, and CFO crashes. Considering the day of the week, the ORs from Tuesday, Wednesday, and Friday were all significantly higher than that on Monday for older drivers involved in CMVT crashes while the ORs for CMVT crashes on Thursday and Sunday were lower than on Monday. Unlike CMVT crashes, most of the ORs for CSV crashes were lower than the reference Monday. As for SCP crashes, the ORs on Tuesday, Saturday, and Sunday were significantly higher than that of the reference status (i.e., Monday). As for the influence of vehicle types, older truck drivers had higher ORs for CMVT and CSV crashes but lower OR for CFO crashes than passenger car drivers. In contrast to the younger and middle-aged drivers, older bus and truck drivers had a higher involvement in CSV crashes than passenger car drivers. The road type results of older drivers show that the ORs for CSV, SCP, and CFO crashes on medium- and high-speed limit roads were lower than on low-speed limit roads, but the OR for CMVT crashes on high-speed limit roads was significantly higher (OR = 30180.68, *p* < 0.001) than on low-speed limit roads. 

### 3.4. General Comparison of Drivers’ Crash Involvement Between Different Age Groups

For a general overview of the differences of drivers’ crash involvement between different age groups, we used the younger group as the reference and examined the ORs of the middle-aged and older groups for different crash types. The results are shown in Table 6. The ORs of middle-aged and older drivers for CMVT and SCP crashes were all significantly lower than the reference younger drivers, while the opposite trend was observed for the CFO crashes. To compare the results before and after using SMOTE + ENN, we further examined the ORs using the extremely imbalanced original data and the results are included in Table 7. By comparing the results shown in Table 6 and Table 7, a significantly higher OR for middle-aged drivers than for the reference younger group was observed for OCV crashes. Different from the results shown in Table 6, the OR of middle-aged drivers for CMVT crashes was higher than 1.00 and the OR for CFO crashes was lower than 1.00. The OR results of older drivers for CFO crashes in Table 6 and Table 7 were also different. Besides, more significant results of older drivers were observed after using SMOTE + ENN (i.e., CMVT: OR = 0.68, *p* < 0.001; SCP: OR = 0.55, *p* < 0.001).

Previous studies have shown that logistic regression is sensitive to imbalanced data [29,30], and the results will become less reliable when there is a large group imbalance problem in the examined dataset [32]. To examine how well a model can explain the data, Nagelkerke *R*^2^ square was frequently used as a quantitative index for evaluation [33]. Its value is in the range of [0, 1] and a larger value means a better fitting of the model. In this study, the Nagelkerke R squares of the older drivers’ models before using SMOTE + ENN were 0.050, 0.064, 0.163, 0.091, and 0.077 for CMVT, CSV, OCV, SCP, and CFO crashes, respectively. The numbers increased to 0.773, 0.413, 1.00, 0.650, and 0.693 for the five crash types respectively after using SMOTE + ENN, indicating that the reported results after using SMOTE + ENN were more reliable.

## 4. Discussion

As reported in the results from Table 3, Table 4 and Table 5, female drivers were more likely to be involved in SMVT crashes than male drivers in all three age groups. At the same time, the OR of CMVT crashes was substantially higher in older female drivers than those in younger and middle-aged drivers. This could be attributed to the fact that male drivers have higher driving skills in handling complex driving situations than female drivers [34]. Laapotti et al. [34] also found that female drivers drove less than male drivers. The less driving exposure time and higher involvement in CMVT crashes indicates the necessity of developing effective solutions to enhance driving safety and skills for female drivers, especially for older female drivers. Kim et al. [35] found that male drivers had a higher probability of being involved in crashes with fixed objects. Our results showed the same trend for middle-aged and older drivers, but the results for younger drivers did not show a significant gender effect. Given the age-related and gender-related differences observed in the present study, future studies should consider both factors when investigating drivers’ crash involvement characteristics based on big crash records data.

Our results showed that weather influenced drivers’ crash involvement, but its effects were different across the examined age groups. Most crash types were more likely to occur on sunny days than on rainy days for younger and older drivers, but the situation was different for middle-aged drivers. Younger and older drivers would prefer not to travel on rainy days (especially in heavy rain) for safety reasons, and the reduced travelling frequency on rainy days would lead to the lower number of crashes on rainy days [36]. This result is consistent with a previous study that reported drivers were more likely to be involved in crashes on rainy days [17]. For older drivers, the higher OR in CMVT crashes on rainy days could be further explained in the way that older adults have degraded visual and visual-cognitive functions, so a rainy environment that is often associated with decreased visibility would make it more difficult for older drivers to detect moving vehicles in transport [37]. For younger drivers, the higher OR in CFO crashes on rainy days is because of their lack of driving experience and skills. A similar trend of crash involvement for younger drivers was also reported in [12]. For middle-aged drivers, the higher ORs in OCV, SCP, and CFO crashes on rainy days could be attributed to their underestimation of hazards and low levels of motivation to follow traffic rules [34]. Snowy weather was not examined in this study because there is no snow in Shenzhen.

Another interesting finding from this study is that time of day and day of the week affected the drivers’ involvement in different crash types which is consistent with the findings in [38], but their effects appeared to be different between the three age groups. Our results on time of day show that drivers were all less likely to crash with pedestrians at midnight in all three age groups because of less pedestrian activity at midnight. However, different from the middle-aged drivers, younger drivers were likely to crash with pedestrians during the period between 18 pm and 23 pm. A reason may be that younger drivers lack driving experience in dealing with complex driving scenarios at night [39]. Another reason is that younger drivers are more likely to experience alcohol driving at night, which has been widely accepted to degrade drivers’ situational awareness for environment perception [40]. Meanwhile, the complex illumination (e.g., oncoming car lights) and low reflection (e.g., pedestrians wearing black clothes) could also increase the probability of pedestrian-related crashes at night. The results reported in [29] confirm that time of day is associated with crash risk, but the differences between different crash types have not been investigated for drivers with different ages. Besides, older drivers’ involvement in SCP crashes on weekdays was also different from that of younger and middle-aged drivers, which may be attributed to the fact that older drivers had different travel patterns in their retirement [41,42]. The authors of [21] reported that crash risk was the greatest at night on weekends, which is consistent with the SCP results on Sunday for the middle-aged and older drivers.

As for the influence of vehicle type, our results show that truck drivers from all three age groups had a higher risk of crashing with moving vehicles in transport than car drivers in the same age group, which is consistent with the results reported in [43] that younger heavy vehicle drivers had higher rates of accident involvement. Normally, truck drivers are not able to see the whole surrounding area of the vehicle due to large blind spot regions [44]. Moreover, truck drivers usually work with fatigue which is one of the most important causes for traffic crashes [45]. Due to the existence of these factors, it was hypothesized that truck drivers would be more likely to be involved in crashes of any type than car drivers regardless of their ages. However, our results did not support this hypothesis. More detailed investigations are needed to further explore the causations of truck crashes.

Considering the influence of road type on drivers’ crash involvement, it was found that drivers from all three age groups were less likely to be involved in CSV crashes while driving on medium-speed limit and high-speed limit roads. This is because there are far fewer static vehicles (usually parked vehicles) on medium-speed limit and high-speed limit roads than on low-speed limit roads. In contrast to middle-aged and older drivers, younger drivers were more likely to be involved in crashes with fixed objects on medium-speed limit and high-speed limit roads. Because of lack of experience, younger drivers usually have a longer reaction time than middle-aged drivers [46]. Unlike in the low-speed limit situations where fast reaction is less critical for safe driving [47], reaction delays while driving on medium-speed limit or high-speed limit roads would definitely increase the risk of crashes. Therefore, the shorter reaction time of experienced middle-aged drivers led to the lower involvement levels in crashes with fixed objects on medium-speed limit and high-speed limit roads than younger drivers.

As given in Table 2, the numbers of CSV, OCV, SCP, and CFO crashes were very low, especially when the numbers were divided into different subgroups (e.g., Monday to Sunday in the day of the week). The extremely low numbers of crashes for older drivers would result in unreliable results, hence the widely accepted SMOTE+ENN method was used to solve the data imbalance problem. In general, the SMOTE method upsamples the older and middle-aged groups for more data and the ENN method downsamples all the age groups for data cleaning [26,27,28]. By comprehensively using SMOTE and ENN, a balanced dataset could be obtained for further analysis. As presented in Table 6 and Table 7, some of the results before using SMOTE + ENN were adjusted, and more statistical significances were observed after using SMOTE + ENN. For deeper investigation into traffic crashes, more crash records should be collected to further examine the findings in this study for more reliable results.

It has been accepted that drivers’ age influences their driving behavior and crash involvement [48,49]. However, previous literature did not report whether the influence of age on crash involvement differed across different types of crashes. The present study has at least partly addressed this problem. Though some similar crash involvement patterns were observed in different age groups (e.g., female drivers were more often involved in CMVT crashes compared to male drivers), drivers with different ages were apparently involved in most crashes in different ways. Specifically, CMVT crashes were less likely to take place on rainy days than on sunny days for younger and middle-aged drivers. However, the situation was quite different for older drivers. Other crash involvement differences between age groups include drivers’ higher involvement in CFO crashes on rainy days than on sunny days for the younger and middle-aged groups but not for the older group; drivers’ higher involvement in SCP crashes on medium-speed limit roads than on low-speed limit roads in the younger group but not in the middle-aged and older groups, etc. These differences should be considered in the development of countermeasures for driving safety enhancement.

It should be noted that this study was based on police-reported crashes. However, the possibility of under-reporting of severe crashes may diminish the reliability of police reports [50]. A comparison study showed that the number of crash fatalities reported from the Chinese Center for Disease Control and Prevention was about twice the police-reported number [51]. Therefore, we would suggest the integration of police-reported data and other data sources (e.g., emergency medical center, forensic institution) to obtain more comprehensive results in future studies.

With respect to crash causation, “other unsafe driver behavior while driving” ranks the highest, accounting for 53.2% of all the crashes and 58.5% of all the deaths in the three-year dataset of this study. This causation covers driver distraction, drowsy driving, drunk driving, driving on call, pedestrian or cyclist not following traffic rules, etc. However, the exact detailed causations were not recorded by the polices. Meanwhile, the exposure information (e.g., vehicle kilometers) was also not recorded for the crashes and the numbers of driving licenses in different age groups were not available to the public. To further improve the quality of police-reported crash records for driving safety enhancement in Shenzhen, traffic police should clearly specify the detailed causations and exposure in the crash records.

The main limitations of this study are the lack of older drivers’ crash record data and the unavailability of the driving exposure information. In our future work, measures should be taken to include more crash records of older drivers and driving exposure data in analysis so as to improve our understanding of the impacts of these factors. Besides, future work should also focus on the integration of police-reported data and other data sources (e.g., emergency medical center, forensic institution) to obtain more comprehensive results. Moreover, the causations (e.g., speeding, distraction, driving under the influence of alcohol) of drivers’ involvement in crashes with different severities for different age groups and the association between crash severity and driver age should be further studied for more in-depth investigations based on a dataset with complete crash records. As the rear-end, head-on, and intersection related crashes are common types in CMVT crashes, separate analysis on these specific crashes should also be conducted in future studies.

## 5. Conclusions

Based on the three-year police-reported crash data in Shenzhen, China, this study examined the crash involvement of drivers from different age groups. The results showed that drivers’ involvement patterns in different crash types were affected by different factors, and the involvement patterns differed among the examined age groups. For example, CMVT crashes were less likely to take place on rainy days than on sunny days for younger and middle-aged drivers, however, the situation was quite different for older drivers. The reported significant differences indicated that the examined factors affected crash occurrence in different ways among the crash types for different age groups, indicating that individualized systems should be designed for the prevention of different crash types and for drivers from different age groups. This extends the previous work from a single factor analysis or mixed crash type analysis to an analysis on the influencing factors. To the best of our knowledge, the present study is one of the first attempts to examine drivers’ crash involvement in China by considering driver age and crash type. Knowledge generated from the present study could help improve the development of countermeasures for driving safety enhancement.

## Figures and Tables

**Table 1:** Recorded crash attributes.

Attribute	Attribute Status Value	Number of Crashes	Percentage
Age	1: Younger (ref.)	41,298	57.2%
	2: Middle-aged	30,169	41.8%
	3: Older	771	1.1%
Weather	1: Sunny (ref.)	39,879	55.2%
	2: Rainy	32,359	44.8%
Gender	1: Male (ref.)	63,675	88.1%
	2: Female	8563	11.9%
Time of day	1: 6~11 (ref.)	18,130	25.1%
	2: 12~17	25,804	35.7%
	3: 18~23	21,317	29.5%
	4: 0~5	6987	9.7%
Day of the week	1: Monday (ref.)	10,354	14.3%
	2: Tuesday	10,320	14.3%
	3: Wednesday	10,471	14.5%
	4: Thursday	10,328	14.3%
	5: Friday	10,891	15.1%
	6: Saturday	10,447	14.5%
	7: Sunday	9427	13.0%
Vehicle type	1: Car (ref.)	45,980	63.7%
	2: Bus	13,680	18.9%
	3: Truck	7505	10.4%
	4: Others	5073	7.0%
Road type	1: Low-speed limit (ref.)	6133	8.5%
	2: Medium-speed limit	55,904	77.4%
	3: High-speed limit	10,201	14.1%

Ref. = reference (no exposure).

**Table 2:** The number of drivers in each age group for each crash type.

Age Groups	CMVT	CSV	OCV	SCP	CFO
Younger	31,772	600	1598	3388	3329
Middle-aged	23,486	460	1381	2383	2015
Older	606	14	31	65	42

CMVT: crashes with motor vehicles in transport, CSV: crashes with stopped vehicles, OCV: other crashes between vehicles, SCP: sideswipe crashes with pedestrians, CFO: crashes with fixed objects.

**Table 3:** Odds ratio results for younger drivers.

Attribute	Values	CMVT		CSV		OCV		SCP		CFO	
		*p*	OR	*p*	OR	*p*	OR	*p*	OR	*p*	OR
Weather	Sunny (ref.)		1.00		1.00		1.00		1.00		1.00
	Rainy	**0.037**	**0.85**	**0.000**	**0.41**	**0.000**	**0.35**	0.220	1.15	**0.000**	**2.16**
Gender	Male (ref.)		1.00		1.00		1.00		1.00		1.00
	Female	**0.000**	**1.41**	0.842	1.05	**0.028**	**0.16**	**0.000**	**0.49**	0.080	1.27
Time of day	6−11 (ref.)		1.00		1.00		1.00		1.00		1.00
	12−17	**0.000**	**4.30**	0.350	1.54	**0.042**	**1.23**	**0.000**	**0.23**	**0.000**	**0.20**
	18−23	0.583	1.06	0.104	2.08	0.956	1.95	**0.001**	**1.72**	**0.000**	**0.32**
	0−5	0.641	0.94	**0.000**	**10.13**	**0.000**	**1.02**	**0.008**	**0.59**	0.197	0.82
Day of the week	Monday (ref.)		1.00		1.00		1.00		1.00		1.00
	Tuesday	**0.000**	**2.67**	**0.000**	**0.28**	**0.000**	**0.26**	**0.005**	**0.51**	**0.033**	**0.65**
	Wednesday	**0.001**	**0.66**	0.310	0.72	**0.000**	**0.25**	**0.000**	**4.51**	0.403	0.84
	Thursday	0.067	1.29	**0.001**	**0.24**	**0.000**	**0.21**	**0.001**	**1.96**	0.189	1.30
	Friday	**0.010**	**1.43**	**0.004**	**0.38**	**0.000**	**0.07**	**0.000**	**2.29**	**0.045**	**0.64**
	Saturday	**0.004**	**1.45**	**0.017**	**1.89**	**0.000**	**0.23**	0.225	0.77	0.054	0.67
	Sunday	0.097	1.24	**0.000**	**0.26**	**0.000**	**0.08**	0.083	1.42	**0.022**	**1.54**
Vehicle types	Car (ref.)		1.00		1.00		1.00		1.00		1.00
	Bus	**0.000**	**1.40**	**0.030**	**0.58**	**0.003**	**0.41**	0.161	0.81	0.270	0.86
	Truck	**0.000**	**1.54**	0.742	1.09	**0.001**	**0.43**	**0.000**	**0.20**	0.740	1.06
	Other	**0.000**	**0.53**	**0.026**	**0.20**	**0.000**	**0.04**	**0.000**	**5.84**	**0.000**	**0.06**
Road types	Low-speed limit (ref.)		1.00		1.00		1.00		1.00		1.00
	Medium-speed limit	**0.000**	**0.58**	0.096	0.66	**0.033**	**1.96**	**0.000**	**2.29**	**0.046**	**1.42**
	High-speed limit	**0.000**	**0.52**	**0.032**	**0.48**	**0.000**	**0.29**	**0.000**	**0.26**	**0.000**	**5.50**

CMVT: crashes with motor vehicles in transport, CSV: crashes with stopped vehicles, OCV: other crashes between vehicles, SCP: sideswipe crashes with pedestrians, CFO: crashes with fixed objects. The bold numbers indicate that statistical significances were observed.

**Table 4:** Odds ratio results for middle-aged drivers.

Attribute	Values	CMVT		CSV		OCV		SCP		CFO	
		*p*	OR	*p*	OR	*p*	OR	*p*	OR	*p*	OR
Weather	Sunny (ref.)		1.00		1.00		1.00		1.00		1.00
	Rainy	**0.000**	**0.41**	0.361	1.17	**0.005**	**1.47**	**0.011**	**1.30**	**0.000**	**2.64**
Gender	Male (ref.)		1.00		1.00		1.00		1.00		1.00
	Female	**0.000**	**1.80**	**0.000**	**0.21**	**0.000**	**0.73**	0.860	0.98	**0.000**	**0.50**
Time of day	6−11 (ref.)		1.00		1.00		1.00		1.00		1.00
	12−17	**0.045**	**1.18**	0.375	0.78	**0.000**	**1.11**	0.071	1.24	**0.001**	**0.70**
	18−23	**0.003**	**1.35**	**0.000**	**6.48**	**0.010**	**0.43**	**0.000**	**0.47**	**0.030**	**0.75**
	0−5	0.356	0.89	**0.000**	**3.33**	**0.000**	**0.45**	**0.000**	**0.31**	**0.000**	**1.66**
Day of the week	Monday (ref.)		1.00		1.00		1.00		1.00		1.00
	Tuesday	**0.000**	**0.13**	0.564	1.20	**0.000**	**2.91**	**0.000**	**14.44**	**0.000**	**2.82**
	Wednesday	**0.000**	**0.16**	0.822	0.92	**0.048**	**4.40**	**0.000**	**8.19**	**0.000**	**2.75**
	Thursday	0.628	0.94	**0.010**	**0.39**	**0.000**	**1.66**	**0.001**	**2.10**	0.091	0.74
	Friday	**0.000**	**0.17**	**0.000**	**2.99**	**0.001**	**14.67**	**0.000**	**5.90**	0.410	1.18
	Saturday	**0.000**	**0.23**	0.604	1.18	**0.000**	**2.52**	0.125	1.50	**0.000**	**4.50**
	Sunday	**0.000**	**0.07**	**0.000**	**5.48**	**0.000**	**6.60**	**0.000**	**11.59**	**0.000**	**3.91**
Vehicle types	Car (ref.)		1.00		1.00		1.00		1.00		1.00
	Bus	**0.000**	**1.90**	**0.000**	**0.31**	**0.000**	**0.61**	0.102	1.21	**0.000**	**0.55**
	Truck	**0.000**	**1.49**	**0.002**	**0.44**	**0.000**	**0.15**	**0.039**	**1.35**	0.678	0.95
	Other	**0.000**	**1.56**	0.981	0.99	**0.000**	**0.32**	**0.000**	**0.19**	0.287	1.15
Road types	Low-speed limit (ref.)		1.00		1.00		1.00		1.00		1.00
	Medium-speed limit	0.441	1.08	**0.005**	**0.58**	**0.000**	**3.49**	**0.002**	**0.67**	0.282	0.87
	High-speed limit	**0.000**	**3.70**	**0.000**	**0.09**	**0.000**	**0.07**	**0.000**	**0.09**	0.541	1.09

CMVT: crashes with motor vehicles in transport, CSV: crashes with stopped vehicles, OCV: other crashes between vehicles, SCP: sideswipe crashes with pedestrians, CFO: crashes with fixed objects. The bold numbers indicate that statistical significances were observed.

**Table 5:** Odds ratio results for older drivers

Attribute	Values	CMVT		CSV		OCV		SCP		CFO	
		*p*	OR	*p*	OR	*p*	OR	*p*	OR	*p*	OR
Weather	Sunny (ref.)		1.00		1.00		1.00		1.00		1.00
	Rainy	**0.000**	**67.62**	**0.000**	**0.27**	0.909	0.00	**0.000**	**0.03**	**0.000**	**0.10**
Gender	Male (ref.)		1.00		1.00		1.00		1.00		1.00
	Female	**0.000**	**60.47**	0.991	0.00	1.000	4.25E + 38	0.992	0.00	**0.000**	**0.03**
Time of day	6−11 (ref.)		1.00		1.00		1.00		1.00		1.00
	12−17	**0.000**	**0.19**	**0.000**	**7.20**	0.988	0.00	**0.000**	**2.84**	**0.001**	**1.38**
	18−23	**0.022**	**1.69**	**0.000**	**0.10**	0.996	3.78E + 11	0.101	1.57	**0.000**	**0.11**
	0−5	**0.000**	**2.78**	**0.000**	**4.05**	1.000	0.00	**0.000**	**0.05**	**0.000**	**0.18**
Day of the week	Monday (ref.)		1.00		1.00		1.00		1.00		1.00
	Tuesday	**0.002**	**1.89**	**0.000**	**0.25**	0.973	1.24E + 27	**0.000**	**4.23**	**0.000**	**0.17**
	Wednesday	**0.000**	**2.62**	**0.000**	**0.34**	0.999	1.28E + 15	**0.000**	**0.17**	**0.020**	**1.56**
	Thursday	**0.000**	**0.01**	0.473	0.83	0.995	0.17	0.991	0.00	**0.000**	**6.69**
	Friday	**0.000**	**9.66**	0.977	0.00	0.971	0.00	**0.000**	**0.02**	**0.000**	**0.36**
	Saturday	0.908	1.03	**0.000**	**0.26**	0.990	8.88E + 14	**0.000**	**4.25**	**0.000**	**0.12**
	Sunday	**0.000**	**0.24**	**0.000**	**0.09**	0.999	1.58E + 42	**0.000**	**15.56**	**0.000**	**0.03**
Vehicle types	Car (ref.)		1.00		1.00		1.00		1.00		1.00
	Bus	**0.000**	**0.02**	**0.000**	**11.44**	0.935	0.00	**0.000**	**28.11**	**0.000**	**0.33**
	Truck	**0.000**	**2.97**	**0.000**	**292.22**	0.897	0.00	0.994	0.00	**0.000**	**0.22**
	Other	**0.000**	**55.02**	0.494	1.19	0.994	0.00	**0.000**	**14.02**	0.977	0.00
Road types	Low-speed limit (ref.)		1.00		1.00		1.00		1.00		1.00
	Medium-speed limit	0.265	0.85	**0.000**	**0.30**	0.999	2.69E + 25	**0.000**	**0.27**	**0.000**	**0.37**
	High-speed limit	**0.000**	**30,180.68**	**0.001**	**0.04**	0.989	7.79	**0.000**	**0.00**	0.988	0.00

CMVT: crashes with motor vehicles in transport, CSV: crashes with stopped vehicles, OCV: other crashes between vehicles, SCP: sideswipe crashes with pedestrians, CFO: crashes with fixed objects. The bold numbers indicate that statistical significances were observed.

**Table 6:** Odds ratio results for different crash types between the examined age groups based on the data after using SMOTE+ ENN.

Age Groups	CMVT		CSV		OCV		SCP		CFO	
	*p*	OR	*p*	OR	*p*	OR	*p*	OR	*p*	OR
younger (ref.)		1.00		1.00		1.00		1.00		1.00
middle-aged	**0.001**	**0.86**	0.795	1.03	**0.000**	**1.77**	**0.003**	**0.82**	**0.000**	**1.27**
older	**0.000**	**0.68**	0.257	0.89	0.832	1.02	**0.000**	**0.55**	**0.000**	**2.70**

CMVT: crashes with motor vehicles in transport, CSV: crashes with stopped vehicles, OCV: other crashes between vehicles, SCP: sideswipe crashes with pedestrians, CFO: crashes with fixed objects. The bold numbers indicate that statistical significances were observed.

**Table 7:** Odds ratio results for different crash types between the examined age groups based on the data before using SMOTE+ENN.

Age Groups	CMVT		CSV		OCV		SCP		CFO	
	*p*	OR	*p*	OR	*p*	OR	*p*	OR	*p*	OR
younger (ref.)		1.00		1.00		1.00		1.00		1.00
middle-aged	**0.003**	**1.06**	0.433	1.05	**0.000**	**1.19**	0.138	0.96	**0.000**	**0.82**
older	0.196	1.13	0.399	1.26	0.674	1.08	0.798	1.03	**0.004**	**0.63**

CMVT: crashes with motor vehicles in transport, CSV: crashes with stopped vehicles, OCV: other crashes between vehicles, SCP: sideswipe crashes with pedestrians, CFO: crashes with fixed objects. The bold numbers indicate that statistical significances were observed.
